# Air pollution exposure, health and performance in elite female soccer players

**DOI:** 10.3389/fspor.2025.1588093

**Published:** 2025-11-25

**Authors:** Adam Beavan, Ross Julian, Falk Gogolla, Sascha Härtel, Michael Stephen Koehle

**Affiliations:** 1 Science & Innovation, TSG Hoffenheim, Zuzenhausen, Germany; 2 Department of Neuromotor Behavior and Exercise, Institute of Sport and Exercise Sciences, University of Münster, Münster, Germany; 3 TSG ResearchLab GGmbH, Zuzenhausen, Germany; 4 Department of Food Informatics, and Computational Science Hub (CSH), University of Hohenheim, Stuttgart, Germany; 5 Physiology, TSG Hoffenheim, Zuzenhausen, Germany; 6 Environmental Physiology Laboratory, School of Kinesiology, University of British Columbia, Vancouver, BC, Canada; 7 Division of Sport & Exercise Medicine, University of British Columbia, Vancouver, BC, Canada

**Keywords:** exercise, sport, air pollution, ozone, female athletes, soccer, particulate matter

## Abstract

**Introduction:**

Acute exposure to air pollution negatively affects athletes’ ability to perform at their best, with a more pronounced impact observed in females. Despite this, the representation of female-only cohorts is lacking. This study investigates how exposure to air pollution impacts performance and well-being of an elite female soccer team.

**Methods:**

Comprehensive data on external, internal, and subjective load variables were combined with the concentrations of three major air pollutants (Particulate Matter >10 µm and Oxidant (Ox = Ozone + Nitrogen Dioxide) during soccer training and matches in the 2022–23 season. Linear mixed-effects models assessed performance parameters such as distance ran, heart rate, rating of perceived exertion (RPE), accounting for confounding factors such as weather and menstrual cycle phase.

**Results:**

No significant impairments in the physical or physiological demands of athletes were observed with increasing pollution concentrations, nor did athletes’ well-being appear to be disturbed. However, RPE increased by 0.84 units (*p* < .001) if athletes played soccer when exposed to Ox levels above 98.3 (µg/m^3^). Interestingly, this undesirable increase in RPE was mitigated if athletes were also exposed to high levels of Ox in the seven sessions leading up to this event (−0.5 units, *p* < .001).

**Discussion:**

While objective performance measures remained largely unaffected by moderate levels of air pollution, players subjectively experienced a heightened sense of effort. Additionally, an acclimation effect was observed regarding preceding Ox exposure, whereby accounting for pre-event exposure levels to Ox appeared to mitigate the negative impact of high Ox levels on RPE.

## 1 Introduction

Air pollution has emerged as a critical global concern due to rapid industrialization and urbanization. In Europe, air pollution remains the most significant environmental health risk, where it has a profound effect on the population's health and well-being, particularly in urban areas. For instance, 96% of the urban population was exposed to fine particulate matter (PM) levels that exceeded the health guidelines set by the World Health Organization (WHO). Exposure to concentrations above 5 µg/m^3^ PM led to an estimated 275,000 premature deaths in Europe (1). Additionally, exposure to levels above 10 µg/m^3^ nitrogen dioxide (NO_2_) and 70 µg/m^3^ Ozone (O_3_) resulted in an additional 64,000 and 28,000 premature deaths respectively (1).

Recognizing the substantial threat that air pollution poses to human health, the WHO has significantly lowered their target pollution levels, to which all cities are mandated to adhere (2). Although this reduction is a positive step, several studies have highlighted that exposure to air quality below these revised thresholds can still have detrimental effects on individual health. Of particular concern is the fact that these pollution levels may be negligible during a rested state when breathing rates are minimal, but can escalate during routine daily activities such as walking, cycling, or running (3). This increased uptake of pollutants during physical activity can be attributed to two factors. First, physical exertion elevates the minute ventilation, resulting in an increased inhaled dose of pollutants (4). Secondly, during more intense physical activities, breathing patterns tend to shift from predominantly nasal to predominantly oral, bypassing the natural filtration system of the nasal passage (4). For instance, a study revealed that individuals who engaged in four sets of 15-minute exercise bouts with 15-minute breaks in between experienced a 4.5-fold higher deposition of PM_(0.1)_ in their respiratory tract compared to the control group at rest (5). In real-world scenarios, cyclists commuting to work are particularly vulnerable to air pollution from surrounding vehicles compared to passengers inside cars (6).

Building upon foundational research that measures the impacts of various methods of commuting to work (i.e., walking, cycling, driving etc.) (6–8), sport has emerged as a new proxy for investigating the relationship between physical activity and heightened exposure to pollution. Studies conducted in various countries involving sports such as soccer (9, 10), baseball, football (11) and athletics (4, 12) highlight the concern of playing sports in environments with varying levels of pollution, particularly concerning the elite athletic sub-population. Numerous studies have shown that athletes' performance in their respective sports is significantly impaired when exposed to moderate-to-high levels of pollution, both during training sessions (10) and matches (9, 11, 13). For instance, a study by Beavan and colleagues examined how air pollution exposure affected a team of highly talented adolescent male soccer players throughout an entire playing season, encompassing all training sessions and matches (10). The authors reported that when athletes played soccer in moderate concentrations of air pollutants commonly experienced in high-income countries, they exhibited higher internal loads indicated by increased ratings of perceived exertion (RPE) and heart rate (HR), along with reduced external loads such as less total distance. Moreover, the athletes reported worse subjective ratings of well-being the following morning.

While the current body of research has been effective in highlighting the adverse effects of pollution on elite athletes' performance and well-being, there is a notable gap in the inclusion of female athletes as participants in these studies. To the authors' knowledge, the vast majority of research examining pollution in sports has either focused solely on male participants or included a mixture of male and female athletes. Importantly, studies conducted in individual sports, such as athletics and running (12, 14), have reported that female athletes seem to be more affected by air pollution levels when compared to their male counterparts. Nevertheless, the underlying physiological mechanisms of these gender discrepancies remain unknown. Addressing this research gap is essential for gaining a comprehensive understanding of the impact of air pollution on all elite athletes.

One notion worth exploring is whether the influence of pollution on female athletes varies throughout the menstrual cycle. It has been proposed that endogenous progesterone, particularly prominent during the mid-luteal phase, can increase minute ventilation and maximal exercise response (15). Several studies have reported evidence of menstrual-cycle effects on ventilation at rest (16, 17) and during submaximal exercise (18). This heightened ventilation might increase the inhaled dose of pollutants in the airways and lungs. Nevertheless, it is important to note that conflicting research findings suggest a lack of cycle-phase effect on exercise ventilation (16, 17, 19), leading to ongoing debates in the literature. Such variations in ventilation and oxygen consumption may play a role in greater exercise-induced fatigue. A previous study demonstrated that female soccer players were not able to run as far during the yo-yo test while in the luteal phase when compared to the follicular phase (20). The existing literature supports the notion that ventilatory control is indeed hormonally influenced, underscoring the necessity to consider hormonal status in female athletes. However, due to the diverse effects of hormones during different menstrual cycle phases on various physiological aspects and ultimately performance, it remains unclear if a specific phase is associated with a higher susceptibility to the impact of air pollution.

Further investigations that specifically focus on female athletes can provide valuable insights into how pollution affects genders differently, thereby informing targeted strategies to safeguard the health and performance of elite athletes in high-performance team sports. Therefore, this study aims to replicate the methods used by Beavan and colleagues (10) to measure the exposure of a team of elite female soccer players to air pollution throughout an entire sporting season. Building on recent findings, we hypothesized that air pollution would have a negative impact on the physical performance and well-being of elite female soccer players. Furthermore, we attempted to assess the influence of ambient pollution levels on performance would vary across different stages of the menstrual cycle, using the self-report data that the club used to track menstrual phase.

## 2 Materials and methods

### 2.1 Data sample

During the 2022–23 domestic season spanning from July 2022 to May 2023, comprehensive match and training load data were collected from an elite professional female soccer team based in Hoffenheim, Germany. According to the McKay et al. (2022) classification these athletes would be considered Tier 4 (21). The data collected from each session included global positioning system data (GPS), heart rate (HR), and ratings of perceived exertion (RPE). Additionally, players completed a daily wellness questionnaire, detailed further in section 2.4. The team consisted of 21 players with an average age of 24.2 ± 2.7 years. Specific height and weight information was not recorded due to sensitivity concerns. The team competed at the highest level of German soccer, and was composed of six defenders, nine midfielders, and four forwards. Preliminary results indicated that total distances covered was similar among outfield players, whereas goalkeepers (*n* = 2) were excluded from the dataset due to their distinct physical demands. Any observations where an athlete had a session duration of less than 30 min was removed from the dataset to ensure that athletes sufficiently exerted themselves during a session and to avoid scenarios where players might have had minimal involvement, such as substitutions in matches where players may be exposed to minimal physical exertion.

Collectively, the working dataset comprised 27 matches and 180 training sessions, resulting in a total of 311 match observations and 2,173 training observations. Each observation represents a player's participation in a session, and each player had on average 130.7 ± 33.2 observations. The study was carried out in accordance with The Code of Ethics of the World Medical Association (Declaration of Helsinki) for experiments involving humans. Furthermore, the players provided their informed consent for use of their retrospective performance data, and ethical approval was obtained through the University of British Columbia (H22-02172). No new data were collected as part of this study.

### 2.2 Air quality

We focused on the main pollutants of concern in Germany: PM_10_, O_3_, NO_2_ (22, 23). To obtain air quality data, we sourced hourly monitor readings from the air pollution monitoring system of the German Federal Environment Agency (*Umweltbundesamt*). These readings were extracted from all stations situated near the match stadiums, with an average distance of 6.7 km. Additionally, air pollution data for the training centre were sourced from the nearest station (8.38 km away) for the entire duration of the sessions. Then, the inverse distance-weighted means for the pollutants were calculated from the monitor readings in proximity to the stadiums or training fields (9, 24). The weighting was proportionate to the distance between each monitoring station and the testing site, meaning that stations in closer proximity to the testing site had a larger influence compared to stations further away (24). Moreover, as weather conditions can act as environmental confounders of air pollution (9), we further extracted all available hourly monitor readings for temperature, humidity, air pressure and wind speed during the players’ time on the pitch, provided by the German Meteorological Service (*Deutscher Wetterdienst*). A 40-km cut-off radius around each playing field was used to generate the inverse distance weighted means (9, 10).

In the current dataset, we observed an anti-correlation of −0.67 between hourly O_3_ and NO_2_. This phenomenon is common since NO_2_ acts as both an oxygen donor for O_3_ production, and a temporary O_3_ reservoir (25, 26). To address this issue, we incorporated the variable “Ox” by summing the values of NO_2_ and O_3_ to represent the oxidation products resulting from the interaction between the two pollutants (25, 26).

### 2.3 Soccer fields description

The training ground is situated 18 km away from the nearest city, positioned on the outskirts of a village (Sankt Leon-Rot) with a population less than 14,000. The training ground is surrounded by a forest to the south-west and agricultural fields to the north-east. Furthermore, the match stadiums where athletes competed can be described as outdoor playing fields, with only a portion of the spectator seats under shelter but not the field itself.

### 2.4 Athletic performance parameters

To minimize the risk of Type I errors, a select number of performance variables were chosen as dependent variables from the extensive list of performance parameters.

#### 2.4.1 External parameters

Player tracking units provided GPS parameters (10 Hz, Kinexon, Munich, Germany). Kinexon units are suitable for tracking pertinent team-sport variables, and included: total number of accelerations completed, total distance covered (m), and number of sprints (27). An acceleration was quantified as a change in velocity greater than six m/s^2^ for a minimum duration of 0.5 s (27). A sprint was classified when a player maintained a speed exceeding 22 km/h for a minimum duration of 1 s.

#### 2.4.2 Internal parameters

Kinexon units also provided the average HR throughout the session (bpm). Furthermore, Borg CR-10 RPE (No effort at all: 0 – maximal: 10) was collected after each session.

#### 2.4.3 Wellness questionnaire

The athletes received a daily questionnaire to complete on their mobile phones upon waking. The questionnaire was developed by the club and consisted of questions related to athletes' current state of well-being and menstrual cycle. Athletes were asked to estimate their phase based on a theoretical 28-day cycle (28): 1. What phase of the menstrual cycle are you currently in? [Menstruation (1–6), Post menstruation (7–12), Ovulation (13–15), Post ovulation (16–25), or Pre menstruation (26–28)], 2. Please rate your stress level from 1 (no stress) to 10 (very stressful), 3. Please rate your sleep quality from 1 (good/ calm sleep) to 10 (bad/restless sleep), and 4. Please rate your muscle soreness from 1 (body feels good/no pain) to 10 (muscle soreness/pain).

### 2.5 Statistical analysis

Multiple linear mixed-effect models with R package lvme4, version 1.1–34 (29) were independently run for each performance and wellness parameter. For each parameter, a linear mixed-effects model was run for all possible subsets of model designs (*n* = 120) to identify the most concise and best performing model using the Akaike information criterion (AIC). The models included several fixed factors such as menstrual cycle (five phases), session duration, and weather variables (temperature, humidity, wind speed, and pressure). SubjectID (1|Subject) was entered as a random factor to account for the random variance associated with the repeated measures of the players. Resulting models were grouped by the performance variables and ranked by AIC to select the best adapted model design for each performance variable. Furthermore, due to the inherent inaccuracy of the self-report menstrual phase data, each of the models was also run with a two-phase quantification of menstrual phase (i.e., currently menstruating vs. not currently menstruating).

**Figure 1:**
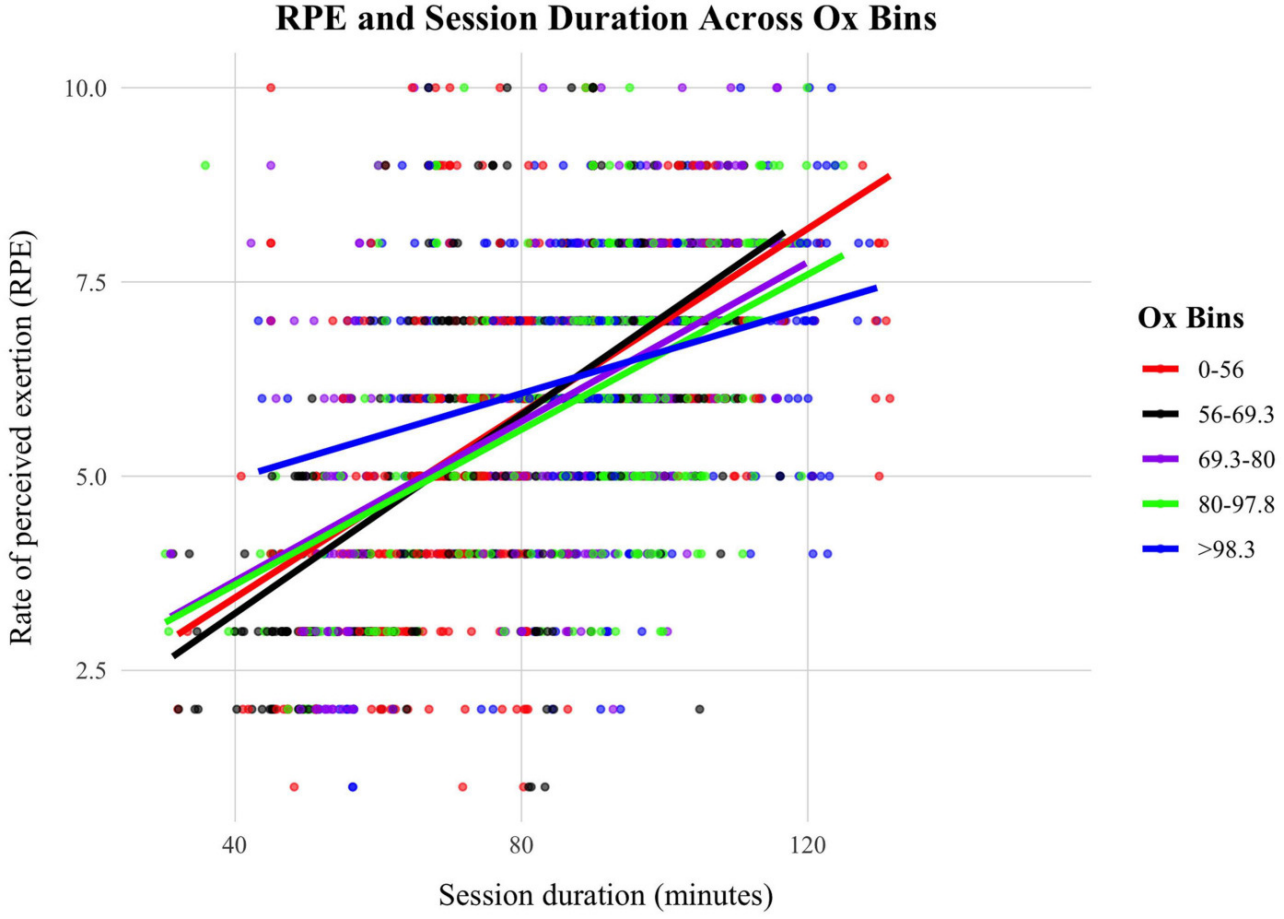
Data visualisation for model A. Model estimates for each of five Ox (µg/m^3^) bins. Coloured circles represent individual data points.

Post analysis, we investigated whether an acclimation effect existed in the variables impacted by air pollution (i.e., RPE). Recent exposure to higher O_3_ levels has been shown to reduce the negative effects of acute exposure of O_3_ on the competitive performances of intercollegiate track and field athletes (30). In accordance with the methods of this paper (30), a seven-session rolling average of O_3_ was created. The rolling O_3_ was then combined with the hourly NO_2_ to create the variable “Rolling Ox”. To explore whether the magnitude of a negative effect on performance is different between low or high recent exposures to O_3_ levels in the time leading up to the competition, Rolling Ox was binned into five exposure levels with approximately equal number of observations (see Table 2). Then, two linear models for RPE were run with all predictor variables included. The only difference between the two models was the inclusion of either the acute exposure of Ox (i.e., no acclimation effect accounted for) or the seven-day rolling average of Ox (i.e., accounting for a possible acclimation effect; see section 3.2.9). Data for model A are visualised in Figure 1.

**Table 2:** A description of Ox (µg/m^3^) bins and model estimates.

**Ox bins (µg/m^3^)** # of observations	0–57.5	57.5–69.5	69.5–80.2	80.2–98.3	>98.3
	477	501	471	524	478
**Model A: not accounting for acclimation effect of O_3_**					
RPE∼Ox (O_3_ + NO_2_) + all fixed factors	Intr	0.14	0.23	0.03	0.84*
**Model B: accounting for acclimation effect of O_3_**					
RPE∼Rolling Ox (Rolling O_3_ + NO_2_) + all fixed factors	Intr	0.05	0.05	−0.18	−0.51*

Model A represent the estimates without accounting for the acclimation effect of O_3_. Model B represents the estimates when accounting for the acclimated effect of O_3_. Intr, Intercept.

* *p* < .001.

## 3 Results

### 3.1 Air quality

Throughout the 2022–23 season, players were exposed to varying levels of air pollutants: PM_10_ (mean: 12.4 ± 7.9 µg/m^3^; range: 3–41.0 µg/m^3^), O_3_ (mean: 67.2 ± 35.4 µg/m^3^; range: 2.8–154.3 µg/m^3^), and NO_2_ (mean: 11.1 ± 12.8 µg/m^3^; range: 1.3–39.6 µg/m^3^). No days were played exceeding the WHO's reference levels of 45 µg/m^3^ for PM_10_, whereas 14% and 4% of sessions were held above 100 µg/m^3^ O_3_ and 25 µg/m^3^ NO_2_ respectively.

### 3.2 Linear mixed-effects models

#### 3.2.1 External load: total distance

The model that best describes the variance in total distance includes all eight parameters, suggesting that total distance is highly dependent on various contextual variables. Among the included parameters, humidity, pressure, and session duration appear to be the most significant parameters influencing total distance (Table 1).

**Table 1:** Summary statistics for the variables included in the models.

Variable	Estimate	Std. error	Wald statistic	*P*-value
RPE∼Ox + Humidity + Wind Speed + Pressure + Menstrual Cycle + Session Duration + (1|SubjectID)				
(Intercept)	−16.026	5.074	−3.158	0.002
Ox (µg/m^3^)	0.007	0.003	2.478	0.013
Session duration (mins)	0.051	0.002	21.704	0.000
Humidity (%)	0.018	0.004	4.524	0.000
Wind speed (m/s)	−0.091	0.026	−3.548	0.000
Pressure (hPa)	0.017	0.005	3.301	0.001
Post menstruation	−0.001	0.128	−0.008	0.993
Ovulation	0.015	0.139	0.110	0.912
Post ovulation	0.031	0.134	0.233	0.816
Pre-menstruation	0.097	0.164	0.591	0.554
RPE∼Rolling Ox + Humidity + Wind Speed + Pressure + Menstrual Cycle + Session Duration + (1|SubjectID)				
(Intercept)	−14.912	5.188	−2.874	0.004
Rolling Ox (µg/m^3^)	0.002	0.003	0.693	0.488
Session duration (mins)	0.053	0.002	21.920	0.000
Humidity (%)	0.012	0.003	3.600	0.000
Wind speed (m/s)	−0.078	0.027	−2.890	0.004
Pressure (hPa)	0.016	0.005	3.104	0.002
Post menstruation	0.034	0.134	0.256	0.798
Ovulation	0.071	0.147	0.487	0.626
Post ovulation	0.035	0.142	0.245	0.807
Pre-menstruation	0.110	0.175	0.628	0.530
Total Distance∼PM_10_ + Ox + Temperature + Humidity + Wind Speed + Pressure + Menstrual Cycle + Session Duration + (1|SubjectID)				
(Intercept)	−21,718.209	5,193.173	−4.182	0.000
PM_10_ (µg/m^3^)	3.665	8.437	0.434	0.664
Ox (µg/m^3^)	−1.903	3.136	−0.607	0.544
Temperature (^o^C)	6.987	8.707	0.802	0.422
Humidity (%)	19.430	4.081	4.761	0.000
Wind speed (m/s)	−24.519	28.232	−0.868	0.385
Pressure (hPa)	20.811	5.155	4.037	0.000
Session duration (mins)	59.999	2.405	24.943	0.000
Post menstruation	−41.240	129.830	−0.318	0.751
Ovulation	−102.926	141.772	−0.726	0.468
Post ovulation	−175.291	135.593	−1.293	0.196
Pre-menstruation	11.051	164.238	0.067	0.946
Session HR∼PM_10_ + Ox + Temperature + Humidity + Wind Speed + Menstrual Cycle + Session Duration + (1|SubjectID)				
(Intercept)	99.432	5.061	19.646	0.000
PM_10_ (µg/m^3^)	−0.222	0.078	−2.841	0.005
Ox (µg/m^3^)	−0.037	0.030	−1.240	0.215
Temperature (^o^C)	0.677	0.083	8.155	0.000
Humidity (%)	0.151	0.039	3.909	0.000
Wind speed (m/s)	−0.618	0.271	−2.281	0.023
Session duration (mins)	0.075	0.023	3.239	0.001
Post menstruation	−0.120	1.242	−0.097	0.923
Ovulation	0.014	1.355	0.010	0.992
Post ovulation	0.501	1.320	0.379	0.704
Pre-menstruation	−2.796	1.580	−1.769	0.077
Accelerations∼PM_10_ + Ox + Temperature + Wind Speed + Menstrual Cycle + Session Duration + (1|SubjectID)				
(Intercept)	1.167	1.864	0.626	0.531
PM_10_ (µg/m^3^)	0.158	0.051	3.074	0.002
Ox (µg/m^3^)	−0.023	0.016	−1.510	0.131
Temperature (^o^C)	−0.378	0.053	−7.107	0.000
Wind speed (m/s)	0.415	0.176	2.366	0.018
Session duration (mins)	0.332	0.015	21.879	0.000
Post menstruation	−0.593	0.819	−0.724	0.469
Ovulation	0.119	0.894	0.134	0.894
Post ovulation	−1.238	0.865	−1.431	0.153
Pre-menstruation	−0.944	1.039	−0.908	0.364
Sprints∼Ox + Humidity + Pressure + Menstrual Cycle + Session Duration + (1|SubjectID)				
(Intercept)	−30.771	8.320	−3.699	0.000
Ox (µg/m^3^)	0.005	0.005	0.979	0.328
Humidity (%)	0.029	0.007	4.387	0.000
Pressure (hPa)	0.028	0.008	3.359	0.001
Session duration (mins)	0.040	0.004	9.886	0.000
Post menstruation	−0.032	0.218	−0.149	0.882
Ovulation	0.010	0.238	0.043	0.966
Post ovulation	0.034	0.230	0.147	0.883
Pre-menstruation	0.076	0.276	0.276	0.782
Stress∼Ox + Temperature + (1|SubjectID)				
(Intercept)	3.640	0.257	14.141	0.000
Ox (µg/m^3^)	0.002	0.002	1.538	0.124
Temperature (^o^C)	−0.032	0.005	−6.221	0.000
Sleep∼Ox + Session Duration + (1|SubjectID)				
(Intercept)	3.740	0.269	13.880	0.000
Ox (µg/m^3^)	0.001	0.002	0.485	0.628
Temperature (^o^C)	0.002	0.006	0.344	0.731
Soreness∼Ox + Session Duration + (1|SubjectID)				
(Intercept)	4.056	0.236	17.155	0.000
Ox (µg/m^3^)	−0.004	0.001	−3.868	0.000
Session duration (mins)	0.016	0.002	9.378	0.000

#### 3.2.2 External load: number of accelerations

The model that best explains the variance in the number of accelerations includes six parameters, indicating again the contextual dependence of this external load variable. Increases in session duration, PM_10_ increased number of accelerations (*p* < .01), whereas increases in temperature reduced the number of accelerations (*p* < .001).

#### 3.2.3 External load: sprints

Five parameters best explained the variance in number of sprints. Increases in humidity, pressure and session duration significantly increased the number of sprints (*p* < .001).

#### 3.2.4 Internal load: average HR

Seven parameters best explained the variance in average session HR, reflecting the significant influence of contextual variables on internal load. Increases in temperature, humidity, and session duration significantly raised session HR (*p* < .001). Conversely, increases in PM_10_ and wind speed significantly reduced session HR (<.02). Notably, being in the pre menstruation phase reduced HR by almost 3 bpm (*p* = .07).

#### 3.2.5 Internal load: RPE

Six parameters best explained RPE. Increases in hourly Ox, session duration, humidity, and pressure significantly elevated self-reported RPE (*p* < .01). Conversely, increases in wind speed decreased RPE (*p* < .001).

#### 3.2.6 Wellness: stress

Only two parameters best explained the variation in stress. Increases in hourly Ox increased stress, yet rising temperature subsequently decreased stress (*p* < .001).

#### 3.2.7 Wellness: sleep

Two parameters also best explained the variation in sleep quality. Increasing hourly Ox (*p* = .63) and temperature (*p* = .73) both negatively impacted sleep quality, but neither reached significance.

#### 3.2.8 Wellness: soreness

Once more, two parameters best explained the variation in muscle soreness. Increases in hourly Ox appeared to improve soreness whereas session duration increased soreness (*p* < .001).

#### 3.2.9 Acclimation effect of Ox on RPE

Table 2 demonstrates that accounting for the recent exposure of O_3_ leading up to the session of interest effectively reversed the significant negative impact of O_3_ on RPE in the highest pollutant bin.

#### 3.2.10 Models using a 2-phase menstrual assessment

**Figure 2:**
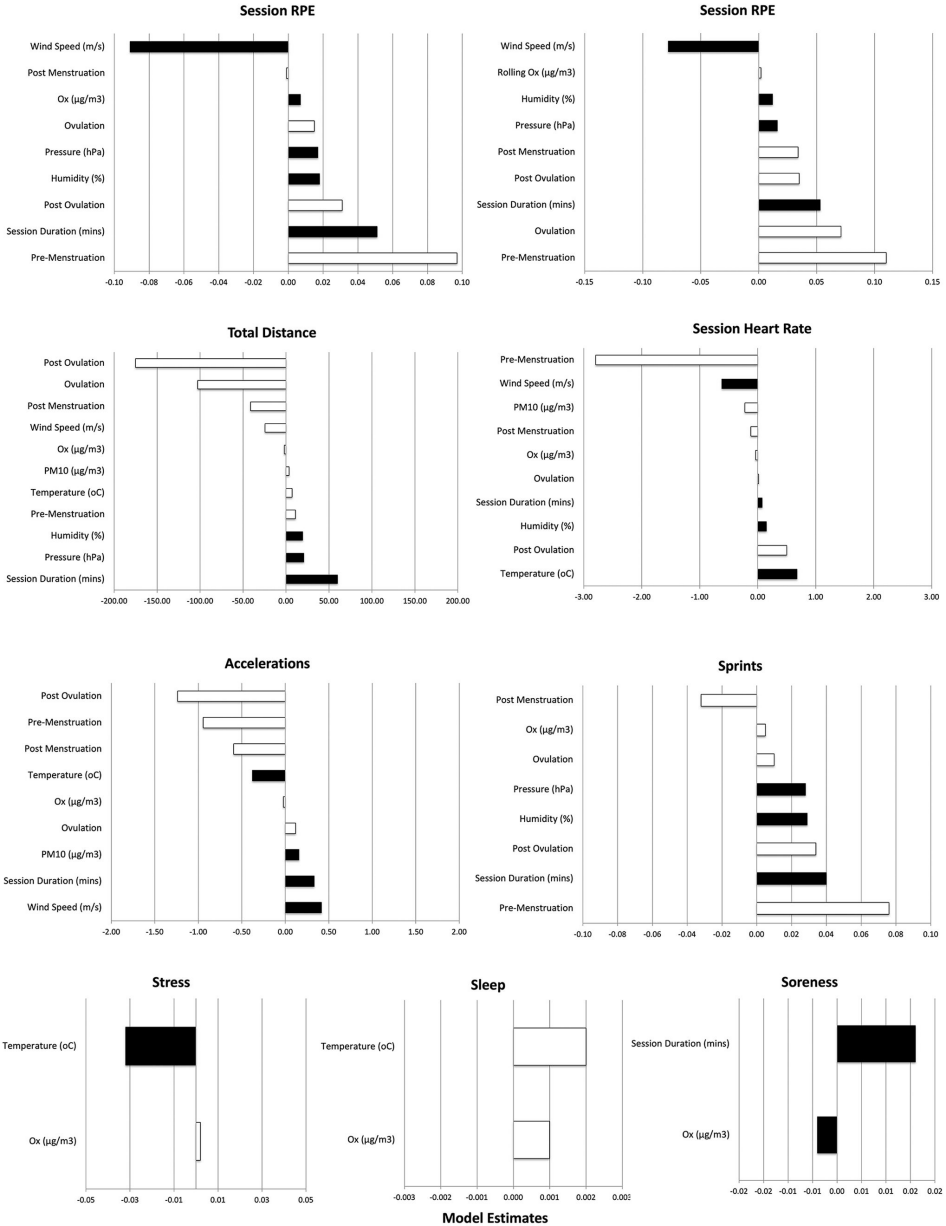
A graphic depiction of each of the models. Significant variables are depicted in black while nonsignificant variables are depicted in white. X-axes represent the magnitude of the estimate for each variable.

All the above models were run substituting a two-phase version of the menstrual self-report (actively menstruating vs., not actively menstruating. For every model, the fit was worse with the 2-phase menstrual assessment. Hence the models presented in Table 1 and Figure 2 represent the 5-phase menstrual assessment.

## 4 Discussion

The current study aimed to investigate whether acute exposure to air pollution influenced the performance and well-being of elite soccer players. It was hypothesised that performance would decrease in relation to increased pollutant concentrations while the athletes were on the field engaging in soccer-related activities. Furthermore, we hypothesised that the relationship between performance and pollution levels might vary across the menstrual cycle. Contrary to our hypotheses, we found no substantial evidence indicating that the pollution levels negatively impacted the players in terms of performance parameters, nor their overall sense of wellbeing. Furthermore, no interaction between the cycle phase and level of pollution on any performance parameter was observed. Nevertheless, the results indicated that athletes' perceived exertion after each session was higher when pollution levels were higher. This suggests that even if the amount of physical exertion an athlete performed remained unaffected, the athletes subjectively experienced an increased sense of effort during sessions conducted under more polluted conditions.

Our findings may seem to contradict the notion that air pollution can impair athletes' performance. However, upon closer examination, our results are consistent with previous studies. For instance, current evidence suggests that athletes begin to experience performance decrements when exposed to PM_10_ levels greater than 20 µg/m^3^ (9, 10). In our study, the majority (88%) of sessions were conducted when PM_10_ levels were below 20 µg/m^3^, with only 11.4% occurring between 20 and 40 µg/m^3^, and a mere 0.3% of all sessions were held at levels exceeding 40 µg/m^3^. Appropriately, we found no evidence that low PM_10_ concentrations impacted player performance. Importantly, only PM_10_ values, but not O_3_ and NO_2_, remained within the WHO guideline values for 2022 across Germany throughout our study (31). Perhaps this explains why Ox was consistently recognized as an influencing factor in all performance and wellness models, signifying its effect across all aspects of performance. Notably, Ox did not have a significant detrimental effect on any performance metric, such as reducing the distance ran or increasing heart rate as reported in previous research (10, 13). Ox was found to only increase athletes' perceived exertion during the session, suggesting that athletes subjectively reported exerting themselves more despite no significant increase in external load. Importantly, this effect only reflected the acute exposure for a single session and does not consider the recent exposure to Ox in the days leading up to this session. A recent field study reported that the effects of contemporaneous O_3_ on performance are moderated by prior exposure to higher levels of ambient O_3_ (30). Additional laboratory studies involving consecutive days of high O_3_ exposure during exercise found a decrease in lung performance during the first three exposure days, but a return to baseline on day four (32, 33). Our study also found evidence of an acclimation effect on O_3_ such that the athletes who had recent exposure to high Ox levels not only mitigated the negative effect of contemporaneous Ox on RPE but actually reported a reduction in RPE of 0.5 units. Therefore, our findings are in line with the position statement by Hung and colleagues, which suggests that exposure to high levels of O_3_ on multiple, consecutive days prior to an event might allow athletes to acclimate to the adverse pulmonary effects of O_3_ exposure (34).

Variations in serum progesterone through the menstrual cycle have been implicated in the effect of air pollution during exercise. Specifically, the decrement in pulmonary function in response to ozone exposure during exercise has been demonstrated to be greater during the follicular phase than during the midluteal phase (35) Through this mechanism, menstrual cycle phase could be important in explaining the variation in external and internal load metrics (36). Our study's ability to assess this question was limited in that it relied on the metric in use by the club which was a self-reported questionnaire in contrast to direct hormonal measurements. We did not find any interaction between air pollution and the menstrual cycle phase in relation to these metrics. Additionally, we found that self-reported menstrual cycle phase did not demonstrate an improvement in the model fit for well-being measures. These findings need to be put into context however, given the limited assessment of menstrual function that was available. The club's routine menstrual cycle assessment only uses questionnaires based on a theoretical 28-day cycle, limiting direct links to sex hormones (28), which are also responsible for physiological changes which may affect the susceptibility to air pollution. These lack of associations are likely more a function of the inadequacy of this tool, and thus further research (including hormonal testing) is warranted to definitively assess the relationship between air quality, menstrual cycle and athletes' capacity to train and perform.

## 5 Limitations

The observational design of the study limits the ability to understand causal factors for the alterations in physical and subject ratings of performance and well-being. However, our field study complements laboratory studies where athletes appear to acclimate to ambient O_3_ and are not impacted by low levels of PM_10_ (34). Our study shares a common limitation with the existing literature, in that we relied on estimated air pollution levels rather than on-site air pollution measurements. Furthermore, we were not able to evaluate the impact of higher levels of air pollution on performance given the lack of observations in poor air quality. According to the air quality index levels reported by the European Environment Agency, 22% of all sessions took place when the air quality was categorized as “good”, 64% as “fair”, 10% as “moderate” and only 5% as “poor”. Additionally, there were some associations that were unexpected with the linear mixed-effects models. Specifically, humidity and pressure were associated with total distance; PM_10_ was associated with an increased number of accelerations; and hourly Ox appeared to improve soreness. Considering the number of performance outcomes that were assessed, Type I error is a possible explanation for these findings, especially in the case of the Ox/soreness association, since the estimate was only −0.004 on a scale of 1–10. Furthermore, for the external load metrics (distance and accelerations), longitudinal external factors such as seasonal changes in weather, air quality and season-long training emphasis could have led to concurrent air quality (PM_10_), weather (humidity and pressure) and session planning trends, which could explain these associations.

## 6 Conclusion

Our study estimated the exposure levels of various air pollutants for an elite female soccer team in Germany across the 2022–23 season. We found that 95% of their sessions were held under good-moderate air quality according to the European Environment Agency's guidelines. We observed no performance-related decrements due to air quality exposure but did find evidence that athlete's RPE was impacted. However, these negative effects of acute exposure to high Ox might be mitigated if athletes were recently exposed to high Ox. In summary, our study provides evidence that teams that are situated in good-fair levels of air quality are not significantly impacted by low levels of pollution.

## Data Availability

The data analyzed in this study is subject to the following licenses/restrictions: the data that support the findings of this study are available on reasonable request from the first author (AB). The data are not publicly available due to their containing information that could compromise the privacy of research participants. Requests to access these datasets should be directed to Dr. Adam Beavan, adam.beavan@tsg-hoffenheim.de.
